# Extracellular Vesicles in Prostate Cancer Carcinogenesis, Diagnosis, and Management

**DOI:** 10.3389/fonc.2018.00222

**Published:** 2018-06-13

**Authors:** Virginie Vlaeminck-Guillem

**Affiliations:** ^1^Medical Unit of Molecular Oncology and Transfer, Department of Biochemistry and Molecular Biology, Centre Hospitalier Lyon-Sud, Hospices Civils of Lyon, Pierre-Bénite, France; ^2^Cancer Research Centre of Lyon, U1052 INSERM, CNRS 5286, Claude Bernard University Lyon 1, Léon Bérard Centre, Lyon, France

**Keywords:** extracellular vesicles, prostate cancer, microenvironment, diagnosis, theranostic, exosomes

## Abstract

Extracellular vesicles (EVs), especially exosomes, are now well recognized as major ways by which cancer cells interact with each other and stromal cells. The meaningful messages transmitted by the EVs are carried by all components of the EVs, i.e., the membrane lipids and the cargo (DNAs, RNAs, microRNAs, long non-coding RNAs, proteins). They are clearly part of the armed arsenal by which cancer cells obtain and share more and more advantages to grow and conquer new spaces. Identification of these messages offers a significant opportunity to better understand how a cancer occurs and then develops both locally and distantly. But it also provides a powerful means by which cancer progression can be detected and monitored. In the last few years, significant research efforts have been made to precisely identify how the EV trafficking is modified in cancer cells as compared to normal cells and how this trafficking is altered during cancer progression. Prostate cancer has not escaped this trend. The aim of this review is to describe the results obtained when assessing the meaningful content of prostate cancer- and stromal-derived EVs in terms of a better comprehension of the cellular and molecular mechanisms underlying prostate cancer occurrence and development. This review also deals with the use of EVs as powerful tools to diagnose non-indolent prostate cancer as early as possible and to accurately define, in a personalized approach, its present and potential aggressiveness, its response to treatment (androgen deprivation, chemotherapy, radiation, surgery), and the overall patients’ prognosis.

## Introduction

Cancer is largely thought to occur as a result of one or several genetic events intrinsically appearing in a cell and providing it with a survival and/or growing advantage. The concept that a malignant tumor only occurs and develops by itself has, however, been challenged for many years. It is now well accepted that environmental factors can be key inducers of both cancer appearance and development (1, 2). By a mirror effect, cancer cells are also able to modify the behavior of the cells that constitute their microenvironment (i.e., the stroma) (3). This continuous interplay between cancer and stromal cells requires specific means of communication including direct cell–cell contacts and secreted factors (growth factors, peptides, etc.) that act *via* paracrine pathways (4). Extracellular vesicles (EVs) more recently proved to be another powerful means used by cells to communicate with each other (3, 5).

The field of EVs is extremely fast-moving (6, 7) (1) because of a clear fashion trend, (2) because of the huge hope that the reliable biomarkers we still need for several pathological conditions including cancers will finally emerge from their exploration, and (3) because it is a relative recent concept. The term “extracellular vesicle” was introduced as a generic term to describe any type of membrane-enclosed particles released by any type of cell (including microorganisms) in the extracellular space (8, 9). This definition covers in fact several terms, sometimes used indifferently in publications and including notably exosomes, microvesicles, microparticles, apoptotic particles, apoptotic bodies, oncosomes, etc. and, in the specific field of the prostate gland, prostasomes (10). Exosomes were first described in the 1980s as a means to recycle the transferrin receptor from the plasma membrane through the endocytic compartments (11–14). They have now become the most studied EV portion because of their strong implication in both physiological and pathological processes including cancers. Thought, at their discovery, as artifacts (15) or as a trash bin for unnecessary and redundant proteins (16), they eventually exhibit a high functional ability: secreted by all kinds of cells, they are able to carry and transfer their proteic, lipidic, and nucleotidic content (the “cargo”) to target cells (5, 8, 9).

In this review, we will focus on the role of exosomes in prostate cancer, but also of other EVs because of the persistent difficulty to distinguish between the different types of EVs (17). This difficulty relies on the criteria used to characterize the diverse EV subpopulations. They can be based on size, density, subcellular origin (in other words biogenesis), function, content (the cargo), membrane markers, etc. (17). And these criteria are deeply influenced by both the orientation of the research team and the methods used to detect and/or characterize the isolated EVs (18, 19). Fundamental research will focus on the biological functions of the EVs: whether the EVs bud from the endosomal compartment (endosomes) or the plasma membrane (ectosomes) is consequently extremely important. This in fact goes back to the question of the specific content of the EVs, which is closely related to the EV biogenesis as will be detailed later. Transfer research, which aims to identify biomarkers, will focus on the tissue or biological fluid where the EVs have to be isolated (for example, prostasomes are only found in prostatic fluid and seminal plasma) and the methods to detect and/or quantify the EVs are a major concern for the researchers. In this regard, a definition of exosomes based on size will be preferentially used when isolating EVs by centrifugation-based protocols, while a definition based on the membrane markers will be put forth when using immunodetection methods. Of course, several motivations can animate the researchers at the same time and several definition criteria and methods are usually used simultaneously or sequentially for a specific research. Furthermore, it is not always of major importance to ascertain which types of EVs have been detected or used at the bench if the goal is to develop a reproducible method that allow reliable detection of EV-derived biomarkers or to demonstrate that a type of cells influence the behavior of another one by the means of EVs Because of these general considerations, it should always kept in mind that despite many efforts there is no real consensual nomenclature of EVs widely used in routine research and that comparisons between the numerous publications dealing with EVs is a huge challenge that should take into account an attentive analysis of the “Material and Methods” part of the papers.

We will first attempt to provide the readers some keys to understand EV classifications according size, biogenesis, and function. We will next explore how studies on EVs shed light on prostate cancer occurrence and progression and at last their potential value as diagnostic, prognostic, or theranostic biomarkers of prostate cancer.

## Biogenesis, Structure, and Functions of Exosomes

**Figure 1:**
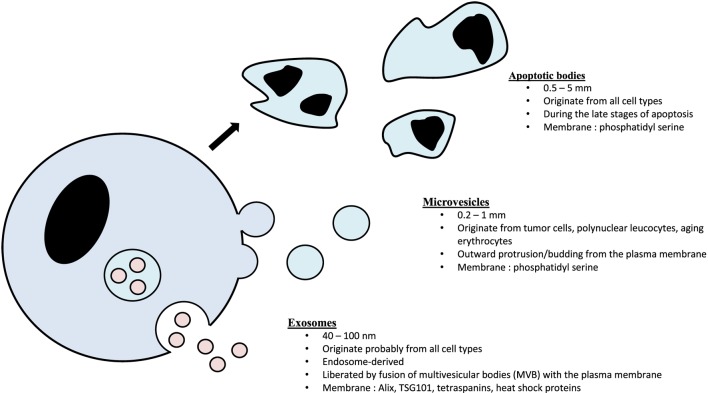
The three main classes of extracellular vesicles.

Exosomes consist of a lipid bilayer membrane trapping a small amount of cytosol that do not contain any organelles (8, 9, 20). Exosomes are classically considered as 30–100 nm EVs originating from intracellular budding from multivesicular bodies (MVBs) (9). They are, therefore, part of the endosomal compartment and strongly differ from the two other main classes of EVs: microvesicles (100–1,000 nm, ectosomal vesicles produced by direct budding from the plasma membrane) and apoptotic bodies (0.5–5 mm, vesicles obtained by cell lysis during the late stages of apoptosis) (21, 22) (Figure 1).

### Exosome Biogenesis, Release, and Capture

The biogenesis of exosomes begins within the endocytic pathway with the formation of late endosomes, the MVBs containing multiple intraluminal bilayered organelles (8, 9). Most of the MVBs fuse to lysosomes and are, therefore, degraded. Some of the MVBs fuse to plasma membrane and release their vesicular content in the extracellular space. The mechanisms by which MVBs are directed toward degradation or fusion to plasma membrane are not well understood. The formation of intraluminal organelles within MVBs has been mostly related to a specific complex of about 30 proteins called endosomal sorting complex required for transport (ESCRT) (8, 9). Specific subpopulations of ESCRT members are responsible for the sequestration of ubiquitinated transmembrane proteins in the endosomal membrane, the membrane deformation into buds and the vesicle scission (23). Accessory proteins, not included in but associated with the ESCRT, are also involved, including ALIX (8). Several studies helped in determining how exosome content is selected and in demonstrating that it is a deliberate, tightly tuned process (9). The membrane constituents of the early endosomes are key elements that govern exosomal content. For example, tetraspanins are proteins of the endosomal membrane able to determine which proteins will be loaded in the intraluminal organelles of the MVBs (9). The lipid composition of the membrane is another major indicator of exosomal content. Likewise, exosome release from parental cells is also governed by specific factors such as the presence of Rab-GTPases, the specific organization of the cytoskeleton and/or the intracellular Ca^2+^ levels (8).

Exosomes are internalized by target cells through different mechanisms including receptor-mediated endocytosis, pinocytosis (invagination of the plasma membrane leading to the formation of a vesicle that contains a more or less high amount of extracellular space), phagocytosis (similar to the pinocytosis but a direct contact is necessary *via* a receptor), and direct fusion with the plasma membrane (8, 9). Like exosome biogenesis and release by parental cells, exosome capture, and internalization by target cells is highly regulated and depends on the local conditions (e.g., microenvironmental pH) (24) and on both the parental and target cell types (9). For example, despite quite similar cargo contents, exosomes derived from the PC3 prostate cancer cell line are taken up by normal epithelial cells at a higher level than LNCaP-derived exosomes (25).

### Exosome Content and Functions

Exosomes contain lipids (essentially in their membranes), proteins, and RNAs (9), or even genomic double-stranded DNA (26). Omics studies in several cell types (including benign and malignant cells) provided exhaustive data about these components, in prostate cancer as an example of all others (25, 27–38) (Table 1). As a consequence of their endosomal origin, exosomes do not exhibit proteins from the nucleus, the Golgi apparatus, the endoplasmic reticulum or the mitochondria. They are rather enriched in proteins from the cytoplasm (including cytoskeletal components such as actin and tubulin), the membrane of the endosomes and the plasma membrane (39). Endosome-associated proteins are Rab-GTPases, SNAREs, annexins, flotillin, and those involved in MVB biogenesis (e.g., ALIX, Tsg101), in exosome sorting (e.g., tetraspanins) or in lipid rafting (e.g., glycosylphosphatidyl inositol-anchored proteins) (9).

**Table 1:** High-throughput studies exploring prostate extracellular vesicles (EVs).

Reference	EV origin	Patients/material	Exosome isolation	Main results
(40)	Human seminal fluid	3 healthy donors	Differential centrifugation	440 proteins identified Including lactoferrin, aminopeptidase N, dipeptidyl peptidase IV, protein-glutamine gammaglutamyl transferase 4, neprilysin

(27)	Cell line from xenografts	PC346C	Differential centrifugation	48 proteins identified Including PDCD6IP, PABC1, EEF1A1, ACTA2, syntenin, GAPDH, LDH-B, FOLH1, ENO1

(41)	Bone metastases	12 mPCa	Differential centrifugation	30 proteins identified Including annexins A2, A3, and A5 and DDAH1

(28)	Cell line	PC3	Differential centrifugation	Microvesicles and exosomes 266 proteins identified involved in transport, cell organization, biogenesis, metabolic processes Including some PC3-specific proteins such as CD151 and CUB domain-containing protein 1
	Plasma	78 men with PCa 28 healthy controls	Ultrafiltration	12 microRNAs (miRs) were differentially quantified in PCa patients compared with controls 11 miRs were present in significantly greater amounts in PCa patients with metastases compared with those without metastases

(42)	Serum	47 men with recurrent PCa 72 men with non-recurrent PCa	ExoMiR extraction kit	miR-141 and miR-375 were associated with recurrent (metastatic) PCa following radical prostatectomy

(43)	Cell lines	PC3 Benign RWPE-1	Differential centrifugation	RWPE-1 cells produce lesser exosomes than PC3 cells miR patterns are not exactly the same in exosomes and in parental cells

(29)	Cell lines	Benign RWPE-1 Androgen-positive LNCaP, C4-2 and VCaPAndrogen negative DU145 and PC3	Differential centrifugation	Some proteins are common to all lines but others are differentially expressed according to malignant status and AR status Proteins associated with malignant cells: ANXA2, CLSTN1, FASN, FLNC, FOLH1, and GDF15 Lipidomics: significant enrichment of exosomes by sphingolipids and glycosphingolipids Increased cholesterol content in malignant cells, without difference for lipid content

(44)	Urine with expressed prostatic secretion	12 LGPCa^a^ 12 men with negative biopsies	Differential centrifugation	Seminal prostasomes and urine exosomes Close to 900 proteins identified

(45)	Human seminal fluid	Healthy donors	Differential centrifugation	In prostasomes, identification of proteins that bound to sepharose-anchored galectin3 Including PSA, PAP, ZAG, CD26, CD13, neprisylin, clusterin, FALL-39, and ORM1

(46)	Urine with expressed prostatic secretion	10 HGPCa^c^ 10 LGPCa^c^ 10 men with negative biopsies	Differential centrifugation	Seminal prostasomes and urine exosomes Specific study of glycans Decrease in larger branched tri- and tetra-antennary glycans in PCa Correlation between the increase in bisecting *N*-acetylglycosamines and PCa severity

(25)	Cell lines	LNCaP PC3	Differential centrifugation	40 proteins common to both cell lines and involved in cell adhesion and migration and cytoskeleton organization 101 proteins overexpressed in PC3 cells as compared to LNCaP cells Including ITGA3, ITGB1, TLN1, and VCL Inhibition of exosomal ITGA3 and ITGB1 using blocking antibodies decreases migration and invasion of normal PrEC cells

(30)	Cell lines	benign PNT2C2 and RWPE-1 malignant PC346C and VCaP	Differential centrifugation	52 proteins differentially expressed between benign and malignant cell lines Including FASN, XPO, PDCD6IP

(31)	Cell line	DU145	Differential centrifugation	Validation of a specific proteomics method (modified aptamer-based Array) 57 proteins overexpressed in exosomes as compared to parental cells

(47)	Cell lines	LNCaP and PC3	Differential centrifugation	AR is present in LNCaP-derived but not PC3-derived exosomes PSMA is present in PCa cell lines and can be used for exosome immunocapture

(32)	Cell line	DU145 with silencing of DIAPH3	Differential centrifugation	Isolation of large oncosomes 407 proteins identified including 103 differentially expressed when comparing with nano-EVs Including CK18 and proteins involved in glucose and glucosamine metabolism, such as GOT1, GAPDH

(48)	Urine	16 men with PCa 15 healthy controls	Differential centrifugation	246 protein differentially expressed Including 37 proteins with potential diagnostic performances (sensitivity >60% at a fixed 100% specificity) Including TM256, LAMTOR1, chr17ORF61, VATL, ADIRF, and Rab-class members

(49)	Human seminal fluid	5 healthy donors	Differential centrifugation	Two distinct prostasome populations according to size and protein content Specific cargo in the two populations: TMPRSS2 was present only in one

(50)	Urine	67 men with PCa 76 men without PCa 13 men after radical prostatectomy 16 women	Time-resolved fluorescence Immunoassay	Increased levels of urinary exosomes after DRE Increased levels of urinary exosomes if PCa

(33)	Primary cell cultures	5 men with PCa	ExoQuick	Identification of exosomes from cells aggregated in bulks and from cancer stem cells 19 miRs differentially expressed in the two cell populations Related to PCa carcinogenesis, fibroblast proliferation, differentiation and migration, angiogenesis and osteoblast differentiation

(34)	Cell lines	LNCaP PC-3	Differential centrifugation	665 proteins identified in LNCaP-derived exosomes 1,735 proteins identified in PC3-derived exosomes

(51)	Human plasma	9 healthy controls 12 men with PCa (4 Afro-American, 4 Hispanic, and 4 Caucasian)	ExoQuick	Increase number of exosomes in PCa Identification of proteins common to all three ethnic groups and ethnic-specific

(52)	Urine	35 men with PCa 35 healthy controls	Lectin-induced agglutination	Analysis of 12 specific miRs miR-574-3p, miR-141-5p, and miR-21-5p were associated with PCa

(53)	Human urine	53 men with PCa 54 healthy controls	Differential centrifugation	11 proteins overexpressed in PCa, including TGM4 and ADSV 3 proteins underexpressed in PCa 45 proteins overexpressed in HGPCa,^b^ including CD63, GLPK5, PSA, PPAP, SPHM

(54)	Human urine	6 men with negative biopsies 12 men with PCa	Differential centrifugation	4,710 proteins identified Including 11 proteins overexpressed in PCa as compared to negative biopsies Including FABP5, granulin, AMBP, CHMP4A, and CHMP4C

(35)	Cell lines	LNCaP LNCaP-C4 LNCaP-C4-2 LNCaP-C4-2B	Immunocapture, size exclusive chromatography, density gradient centrifugation	153 proteins identified Including 8 overexpressed in C4-2B subline as compared to parental LNCaP Including gammaglutamyl transferase (GGT1) overexpressed in C4-2 and C4-2B sublines as compared to LNCaP and C4 subline

(36)	Cell lines	Malignant PC3 cells Benign PNT1A cells	Differential centrifugation	64 proteins exclusively found in PC3-derived exosomes Including claudin 3

(55)	Human seminal plasma	12 healthy donors	30% cushion-based isolation	1,474 proteins identified in prostasomes Involved in metabolism, energy pathways, protein metabolism, cell growth, cell maintenance, transport Including PHGDH, LGALS3BP, SEMG1, ACTB, GAPDH, ALIX

(56)	Urine	4 men with PCa 4 healthy controls	Differential centrifugation	Different lipid species in PCa patients The lipid cargo is different whether EVs are < or >150 nm

(57)	Human urine	3 men with PCa 3 healthy controls	Differential centrifugation	Differential expression of 55 metabolites

(58)	Urine	15 men with PCa 13 men with BPH	Ultracentrifugation	Demonstration of the potential use of exosomal lipid species in urine as prostate cancer biomarkers Specific combination of lipids species (phosphatidyl serine and lactosylceraminde) can distinguish PCa and BPH patients

(59)	Urine	70 PCa 51 BPH	Differential centrifugation	Differential expression of transcripts between the two conditions Including cadherin 3, which is lower in PCa-derived exosomes Confirmation of the decreased expression of the protein in PCa tissues, linked to genetic and epigenetic alterations

(37)	Cell lines	Malignant LNCaP, VCaP, DU145, and PC3 Benign PNT2	Differential centrifugation	Specific exploration of miRs Specific signatures were observed for each of the malignant cell lines Exosomal expression was highly correlated to miRs’ expression in parental cells

(37)	Cell lines	Malignant LNCaP, VCaP, DU145, and PC3 Benign PNT2	Differential centrifugation	Specific exploration of long non-coding RNAs 26 lncRNAs are common to malignant cells comparing to the benign ones Highly enriched in target motif for miRs (these miRs were simultaneously present in exosomes) and RNA-binding proteins

(38)	Cell lines	Malignant LNCaP and PC3 Benign PNT2	Differential centrifugation	Multiplex analyses of 84 genes involved in PCa In both microvesicles and exosomes mRNA content is higher in microvesicles than in exosomes differential expression when comparing benign to malignant cell lines differential expression when comparing the two malignant cell lines

*BPH: benign prostate hypertrophy; HGPCa: high-grade PCa; LGPCa: low-grade PCa; mPCa: metastatic PCa; PCa: prostate cancer*.

*^a^In this study, low grade is defined as Gleason score ≤ 6 and high grade as Gleason score ≥ 7*.

*^b^In this study, low grade is defined as Gleason score ≤ 7 (3 + 4) and high grade as Gleason score ≥ 7 (4 + 3)*.

*^c^In this study, low grade is defined as Gleason score ≤ 6 and high grade as Gleason score ≥ 8*.

As compared to plasma membranes, exosomal membranes are enriched in cholesterol, sphingomyelin, and hexosylceramides, while being bald in phosphatidylcholine and phosphatidylethanolamine. This accounts for the exosome formation within MVBs (60).

A turning point has in fact been marked with the demonstration that exosomes contain mRNAs and microRNAs (miRs) that can be eventually translated into functional proteins upon capture by target cells (5, 61). In other words, exosomes can be considered as intercellular messengers able to transmit biologically active messages from parental cells to target cells (5, 62). The biological behavior of the target cells can, therefore, be distally modified to provide specific advantages in terms of favorable microenvironment (e.g., production of growth factors, neoangiogenesis, remodeling of the extracellular matrix, etc.) (5). Of note, when transfer of oncogenic material is able to induce transformation of the recipient cells (a typical demonstration of a biological significance!), exosomes are called oncosomes (63). Recent studies have also demonstrated that exosomes also contain other varieties of RNA including small or long non-coding RNAs, structural RNAs, tRNAs, and small interfering RNAs, all species with functional interactive abilities despite the absence of protein coding ability (8, 62).

## The Large Family of Prostate EVs: Prostate Exosomes—Prostasomes—Prostate Large Oncosomes

### Prostasomes

Normal prostate cells have been shown to produce EVs for a long time. It is worthy to note that the first prostate-originating EVs, called prostasomes, were reported in the late 70s as extracellular nanosized membrane-surrounded particles found in seminal plasma (64–66). Whether prostasomes are really exosomes remains matter of debate (67, 68). There are similarities such as stockade in the MVBs, release after MVB fusion with the plasma membrane (69), presence of exosomal markers such as CD9 and CD63 (70), and exchange of biologically significant information (71). Nevertheless, prostasomes are usually bigger than exosomes (50–500 nm, the mean diameter being 150 nm) (72), they can have a multilayer membrane (40, 73), their lipidic content is slightly different (higher concentration of cholesterol and sphingomyelin, and higher cholesterol/phospholipid ratio) (70, 72, 74), and they can contain chromosomal DNA (seldom reported in exosomes) (69, 75). As a normal component of the seminal plasma, prostasomes have been shown to interact with spermatozoa and are strongly involved in male fertility and reproduction (76, 77). They are indeed implicated in spermatozoa motility, capacitation, acrosome reaction, modulation of immunological attacks by the female’s immune cells, antioxidant, and antibacterial capacities (69, 71).

Even if most of the studies exploring EVs in prostate cancer focused on “true” prostate exosomes, it should be kept in mind that prostasomes are also produced by prostate cancer cells (78–81), including metastatic ones (79, 80), using the same processes than normal cells (80). Although demonstration is still lacking, their roles in prostate carcinogenesis could be reminiscent of those observed in human reproduction including escape from the immune system or induction of migration (82). Of note, one study suggested that production of prostasomes in prostate cancer cells is inversely correlated with the Gleason score (83). Other studies demonstrated differential expression in prostasomes derived from normal or cancer prostate cells (44–46, 49, 55).

### Prostate Large Oncosomes

Prostate cancer is the tissue in which a new class of EVs has been described in the late 2000s (84, 85). They have been called large oncosomes because of their size (1–10 µm) and their primitive description from cancer cells. Large oncosomes production results from the shedding of non-apoptotic plasma membrane blebs and can be induced by overexpression of oncoproteins (84) or silencing of the cytoskeletal regulator DIAPH3 (85, 86).

Like exosomes, prostate large oncosomes carry abundant bioactive molecules, including signaling factors implicated in cell growth, cell metabolism, cell motility or RNA processing (84), but their cargo seems specific, at least different from that of the nanosized prostate EVs (32). Proteins differentially expressed are involved in cancer-associated metabolic processes such as glucose, glutamine, and amino acid metabolism. Of interest, cancer cells exposed to large oncosomes but not to exosomes disclosed altered glutamine metabolism, suggesting that large oncosomes exert specific functional abilities (32). This specific content may result from their specific release by cells transitioning to an amoeboid phenotype, a phenotype used by some invasive cancer cells to migrate in the peritumoral space. Amoeboid cells affect an elliptical blebbing morphology, which would favor the scission of large oncosomes. As such, large oncosomes could represent markers of invasive, migrating cancer cells (10).

### Prostate Exosomes

Although distinction with other nanosized EVs is usually not fully achieved, prostate exosomes constitute by far the most studied portion of prostate EVs (10, 67). Large-scale profiling experiments have been widely performed, using various origins such as cell lines, plasma, serum, urine, and different methods depending on the part of the content to be targeted (proteins, lipids, metabolites, RNAs, etc.) (Table 1). Comparisons have been made between different cell lines (for example androgen-sensitive or androgen-independent; aggressive or less aggressive, etc.) or between different clinical conditions (benign prostate hypertrophy, localized cancer, metastatic cancer, castration-resistant cancer, etc.). Quite all studies demonstrate that such specific culture or clinical situations can be related to specific expression patterns, fueling the hope to find valuable biomarkers (see below). Along with other tissues, the resulting datasets have generally been introduced in large databases such as Exocarta, Vesiclepedia, and EVpedia (21, 87, 88).

## Biological Functions of Exosomes in Prostate Cancer

In prostate cancer as in all other tissues (benign or malignant), exosomes (or at least EVs) have been found to be produced by all studied cell types. In other words, they are secreted not only by cancer cells but also by all the cells constituting its microenvironment, i.e., cells from the adjacent normal and malignant epithelium, as well as cells from the tumor stroma, such as fibroblasts, immune cells, and endothelial cells (8). All these cell types can interplay with each other by producing EVs and we here successively present the effects of cancer cell-derived exosomes on stromal cells, the effects of stromal cell-derived exosomes on cancer cells, and then the effects of cancer cell-derived exosomes on adjacent epithelial benign or malignant cells.

### Effects of Cancer Cell-Derived Exosomes on Stromal Cells

#### Cancer-Derived EVs Favor the Occurrence of Activated Cancer-Associated Fibroblasts (CAFs)

For cancer cells, the aim of targeting stromal cells by biologically significant exosomes is clearly to modify the microenvironment to favor local and distant extension (2). One of the major changes during cancer and development is the transition from a simple stroma (with only supportive abilities) to a reactive stroma able to favor cancer cell proliferation and metastatic spread (89). This transition is accompanied by changes in all the stromal components: nature and proportion of immune cells, proteic components of the extracellular matrix, etc. All these modifications have been correlated to prostate cancer differentiation (Gleason score) or survival (90). Among these modifications, a fundamental one is the occurrence of CAFs (91), producing several growth factors (TGF-beta, SDF-1, CXCL14, HIF-alpha, etc.) that target cancer cells (92). Conversely, epithelial cells produce several signals able to stimulate CAF occurrence (TGF-beta, PDGF, FGF2, hedgehog, etc.) (92). Other communication means include exosomes, as demonstrated in several cancers.

In this regard, exosomes derived from the prostate cancer cell lines DU145 and PC3 (and to a less extent the LNCaP) contain TGF-beta that can induce transformation of fibroblasts to myofibroblasts (a feature of CAFs) *via* the activation of the TGF-beta/SMAD signaling (93, 94). Indeed, while LNCaP cells produce exosomes with low levels of TGF-beta, other cell types such as PC3 and DU-145 produce exosomes with high TGF-beta levels. Expression of the transmembrane proteoglycan betaglycan at the exosome surface appears to be necessary for TGF-beta loading in the exosomes (93). This TGF-beta is able to trigger the SMAD3-dependant intracellular signaling pathway of target cells (93). TGF-beta could thus induce both the fibroblastic expression of alpha-smooth actin (SMA; at the protein level)—a key marker of myofibroblastic differentiation—and its filamentous structures (93, 94). Fibronectin containing the alternatively spliced EDA exon, another myofibroblast biomarker, also proved to be influenced by exosomal TGF-beta (94). In addition, exosome-derived TGF-beta triggers an alteration of the immediate peri-fibroblastic microenvironment that is also specific for myoblast differentiation: the formation of a pericellular coat largely composed of hyaluronic acid (93). Other responses classically obtained with soluble TGF-beta were also observed (such as autocrine production of TGF-beta by fibroblasts), but it was suggested that exosomal TGF-beta could exert specific intracellular effects such as increased FGF2 expression (93). Similar results were obtained when normal prostate stromal cells (PrSC) were cocultured with prostate cancer cells (92). Of interest these results obtained from fibroblast cultures could be reproduced using normal and activated stromas obtained from fresh tissues (patients undergoing prostatectomies) (94): normal stromal cells do not express alpha-SMA (but gained this ability upon exposition to exosomal TGF-beta), while cells isolated from reactive stroma exhibit heterogeneous but clear alpha-SMA expression that could not by reinforced by stimulation. To ascertain the importance of exosomal delivery over soluble TGF-beta secretion, the use of Rab27a, a regulator of exosome production, allowed clear demonstration that cells selectively deficient in exosome production could not trigger similar responses (94).

Although the underlying mechanisms are not fully understood, hypoxia in prostate cancers has been associated with an aggressive phenotype and a poor prognosis (95). A recent paper showed that exosomes extracted from LNCaP and PC3 cells cultured in hypoxic conditions induced a CAF phenotype in normal human PrSC (96). Expression of the alpha-smooth muscle actin was indeed found to be enhanced (96). It is likely that the exosomal content is modified under hypoxic conditions since hypoxic prostate cancer exosomes are loaded with a significantly higher amount of triglycerides (97).

It has been well demonstrated that CAFs can be obtained from several cell lineages and not only from normal stromal fibroblasts. For example, mesenchymal stem cells (MSCs) are pluripotent cells, able to differentiate into several connective tissues, including myocytes, neurons, osteoblasts, chondrocytes, adipocytes, and fibroblasts (98). The roles of MSCs include migration into inflammatory or injured tissues and their repair. Intratumoral MSC migration is also considered, notably in prostate cancer (99) with tumor-promoting effects similar to those of cancer-associated stroma. A recent experiment suggests that cancer cell-derived exosomes are able to induce differentiation of MSCs into myofibroblasts (100). Bone marrow MSCs exposed to DU145-derived exosomes despite pro-adipogenic culture conditions underwent differentiation into myofibroblasts with expression of alpha-smooth muscle actin and secretion of both growth factors (VEGF-A, HGF) and extracellular matrix regulators (MMP-1, MMP-3, and MMP-13). These properties allow promotion of both angiogenesis and tumor progression (proliferation, migration, and invasion). Again, this reprogramming effect of cancer-derived exosomes was dependent on the presence of exosomal TGF-beta, while soluble TGF-beta did not produce similar effects (100).

It is worthy to note that the way in which fibroblasts engage upon EV stimulation may not be unequivocal and could depend on the nature of the EVs Pioneer study suggested that large oncosomes extracted from the tumorigenic RWPE-2 cell line can induce CAF migration (101). The same team later showed that these oncosomes harbor sustained AKT1 kinase activity that induced prostate fibroblast reprogramming (102). The reprogramming was molecularly defined by the increased expression of alphaSMA, Il-6, and matrix-metalloprotease 9, without any change in TGF-beta levels, suggesting a provascularization phenotype rather than an epithelial-to-mesenchymal transition (EMT) phenotype (102). In fact reprogrammed fibroblasts were effectively able to stimulate endothelial tube formation *in vitro* and tumor growth in mice (102). In these experiments, activation of stromal MYC was found to be the critical event for fibroblast reprogramming.

#### Cancer-Derived EVs Induce Changes in the Extracellular Matrix

Other changes induced in the stroma by cancer-derived MVs include alterations of the extracellular matrix (96, 103) and promotion of angiogenesis (94, 103). Prostate cancer-derived microvesicles indeed proved to express matrix metalloproteinases (MMP) such as MMP9 (PC3 and LNCaP cells) and MMP14 (PC3 cells only), possibly through stimulation of ERK1/2 phosphorylation (103). MVs released by prostate cancer tissues seem, therefore, able to degrade collagen IV and the basal membrane, suggesting a role in promoting both cancer cell protection from apoptosis (specially anoikis, i.e., the specific apoptotic process that results from the isolation of a migrating cell devoid of cell–cell contacts) and mobility of both fibroblasts and cancer cells (103). Of note, prostasomes extracted from PC3 and LNCaP (as compared to prostasomes extracted from healthy ejaculates) could degrade the extracellular matrix through modifications of their surface proteolytic enzymes: decrease in dipeptidyl peptidase IV and increase in UPa (urokinase plasminogen activator) and cathepsin B were observed (104). Since MMPs are also involved in angiogenesis (96, 103), an effect of MV release by prostate cancer cells on neoangiogenesis is also suggested, and consistent with the proangiogenic (although indirect) effect of cancer cell-derived exosomes observed by Webber et al. (94): they found that myofibroblasts activated by cancer-derived exosomal TGF-beta can modulate the behavior of endothelial cells, leading to more branching and elaborated networks of vessel-like structures connected to neighboring vessels (94). Similar effects on cultured endothelial cell proliferation and migration were observed with MVs derived from DU-145 (105). Likewise, stromal cells cocultured with prostate cancer cells also exhibited an increase in VEGF secretion, suggesting a potential proangiogenic effect (92). By contrast, prostasomes obtained from postvasectomy human semen were unable to influence endothelial cell survival and rather inhibited the development of capillary-like networks (106, 107). This discrepancy between prostasomes and other prostate MVs may result from the prostasome specific lipid content, which proved to be enriched in sphingomyelin and cholesterol (see above), even if sphingomyelin was suggested by others as a key mediator of the proangiogenic effects of DU145-derived MVs (105).

#### Cancer-Derived EVs Favor Immune Escape

Immune cells present in the tumor stroma are involved in the host mechanisms of defense against cancer occurrence and development (9). Lundholm et al. demonstrated that prostate cancer-derived exosomes could help cancer cells to evade the immune responses (108). Using flow cytometry experiments, the authors found that ULBP1-2 and MICA/B, ligands for NKG2D [a membrane receptor expressed at the membrane of natural killer cells (NK) and CD8+ T cells that activate target cell death] are expressed at the surface of cancer-derived exosomes. Bound to the NKG2D receptors present on the NK and CD8+ T cells, these exosomal ligands are able to induce downregulation of NKG2D receptors and consequently to impair the cytotoxic function of immune cells (108). Of interest, these *in vitro* results are consistent with the *in vivo* observations that patients with castration-resistant prostate cancer exhibit a significantly decreased surface expression of NKG2D on circulating NK and CD8+ T cells and that cancer-derived exosomes extracted from plasma or serum of these patients induced NKG2D downregulation in cultured lymphocytes (108). A recent study suggested that prostate cancer-derived exosomes may affect the action of CD8+ T cells, through altering the ability of dendritic cells to interact with them (109). The fact that prostate EVs can protect cells from lymphocyte-mediated lysis was previously observed for prostasomes (110). At a molecular level, it was demonstrated that prostasomes express CD59 (also named membrane inhibitor of reactive lysis), an important regulator of the complement cytotoxicity (111). The transfer of exosomal CD59 from prostasomes to red cells lacking CD59 (rabbit erythrocytes and human erythrocytes from patients with paroxysmal nocturnal hemoglobinuria) indeed proved to induce protection against complement-mediated hemolysis (111). Prostasomes produced by PC3 and DU-145 cells proved to contain high CD59 levels as compared to those derived from LNCaP, normal seminal fluid or normal prostate tissues (112). All these prostasomes could, however, be transferred to red cells and decrease complement-mediated hemolysis (112). Prostate large oncosomes are also able to influence immune response, by suppressing proliferation of human macrophages and peripheral blood mononuclear cells (86). This immune suppressive effect could be related to the reduced expression of Akt1 in these tumor-infiltrating immune cells, in response to the miR-125a present in the EVs produced by cancer cells (86).

#### Cancer-Derived EVs Could Induce Tumor-Like Phenotype in MSCs

Fibroblasts are not the only stromal cell type that undergoes transformation upon exosomal stimulation by cancer cells. Adipose-tissue derived stem cells (ASCs), a subtype of MSCs, can be isolated from a perivascular niche of fat tissue and have the ability to differentiate into multiple cell lineages (113). In an intriguing study, ASCs obtained from the fat of the Retzius space of patients undergoing radical prostatectomy for prostate cancer and selected for their high migratory ability were exposed to the culture media of benign (RWPE-1) and malignant cell lines (PC3 and C4-2B). Transplanted in the flanks of athymic nude mice, ASCs exposed to malignant cell culture media developed subcutaneous nodules with neoplastic histopathological features reminiscent of prostate cancer. Expression of epithelial and prostate cancer-specific biomarkers (CK8, CK5/18, and AMACR) fueled the hypothesis that ASCs can form prostate-like neoplastic lesions upon specific stimulation. Further *in vitro* experiments suggested that prostate cancer cell-derived exosomes are responsible for this prostate tumorigenic mimicry and that the ASC reprogramming is linked to the presence of oncogenic factors within exosomes such as H-*ras*, K-*ras*, other members of the Ras superfamily of GTPases and some miRs (miR-125b, miR-130b, miR155) (113). These experiments suggest that MSCs, such as ASCs, can transform into tumor cells, an ability that has in fact not been fully elucidated (114).

#### Circulating Cancer-Derived EVs Could Prepare the Metastatic Niche

Although the effects of exosomes are usually measured in the close microenvironment of cancer cells, there is now strong evidence that they can be released in several body fluids including the blood, as observed for prostate cancer (115). As such, whether they can participate to the metastatic spread remains to be precisely determined but several recent papers favor this hypothesis. As prostate cancer progresses, osteoblastic bone metastases are frequently observed and many experiments suggest that the bone tropism of prostate cancer cells results from a vicious cycle established between cancer and bone cells (osteoblasts, osteoclasts) (116, 117). While prostate cancer cells supply osteoblastic and osteoclastic factors to bone cells, these cells, thereby activated, in turn supply prostate cancer cells with growth factors (116, 117).

Some recent papers suggest that cancer-derived EVs could both favor osteoblast differentiation, impair osteoclast differentiation and favor prostate mimicry in bone cells. For example, exposition of murine pro-osteoblastic MC3T3-E1 cells to the MVs extracted from the prostate cancer cell lines PC3 and DU-145 proved to facilitate osteoblast differentiation (118). Of note, ETS1, a transcription factor known to be involved in many metastatic processes, can be detected in the MVs of PC3 and DU-145 cells, but not in those extracted from LNCaP cells that failed to facilitate osteoblast differentiation (118). Similarly, when exposed to exosomes extracted from the murine prostate cancer cell line TRAMP-C1, monocytic osteoclast precursors (monocyte cell line RAW264-7 and bone marrow-derived osteoclast precursors) failed to fusion and differentiate into mature multinucleated osteoclasts despite induction by the osteoclastogenic factor RANKL (119). Consistently osteoclastic markers (DC-STAMP, TRAP, cathepsin K, MMP9) were significantly less expressed upon exosome exposition. Another study suggested the same mechanism: high amounts of miR-141-3-p present in the MDA-PCa-2b cells (PCa bone metastasis cell line) can be transferred to osteoblasts where they directly target DLC1, a protein involved in the regulation of Rho GTPases (120). The p38MAPK pathway is thereby influenced, inducing increased proliferation, increased calcium deposition and increased expression of markers of osteoblastic differentiation (120). *In vivo* experiments showed that intravenously injected fluorescent-tagged MDA-PCa-2b-derived exosomes were preferentially captured by bone, where they induced osteoblastic activity (120). Similarly, MDA-PCa-2b cells transfected with miR-141-3-p and injected into mice developed bone metastases that reduced the animals’ survival (120).

Cell plasticity can be defined as the ability of a cell to undergo reversible phenotypic changes during tissue invasion. Epithelial cells are particularly able to adopt plasticity as a means to favor distant progression. Prostate cancer cells for example can transit to a mesenchyme-like phenotype (EMT) like many cancer cells. More specifically, evolution toward neuroendocrine differentiation (NED) (121, 122) and osteomimicry (123, 124) was reported in prostate cancer cells, respectively, as a response to androgen deprivation or radiotherapy, and as a means to favor bone metastasis. Prostate cancer cells expressing bone markers (osteomimicry) better adapt to the bone microenvironment. Conversely, bone cells could also produce prostate-specific biomarkers that could help prostate cancer cell integration into the bone tissue. Renzulli et al. demonstrated that human bone marrow freshly harvested from healthy volunteers can express prostate-specific markers (TMPRSS2, PSA, PSCA, ERG, ETV1, KLK3) when cocultured with 1-cm^2^ wide pieces of human prostate cancers (125). MVs isolated from these prostate cancer specimens provoked similar effects.

### Effects of Stromal Cell-Derived EVs on Prostate Cancer Cells

Studies dealing with the EV-mediated interplays between prostate cancer cells and stromal cells essentially addressed the functional properties of cancer-derived exosomes. Analyses of stroma-derived EVs are dramatically less frequent but provide significant results.

One of the studies showed that stromal fibroblasts produce EVs measuring 1–30 µm in size (126). These EVs contain miR-409 which has been previously shown to promote tumorigenesis, EMT, stemness, and bone metastasis of human prostate cancer (127). Stromal-derived EVs can be efficiently internalized by the prostate cancer cell lines ARCaP_E_ and C4-2B, in which they induce specific changes including decreased expression of miR-409 target genes (Stag2, RSU1, PHC3), increased proliferation, morphological EMT, biochemical EMT (decreased expression of E-cadherin and increased vimentin expression), and tumorigenesis in athymic nude mice (126). Subcutaneous coinjection of ARCaP_E_ cells together with miR-409-expressing stromal fibroblasts targeted development of bigger tumors, with higher proliferative rate, than injection of the sole ARCaP_E_ cells. Expression of miR-409 by ARCaP_E_ cells (naturally devoid of it) in the tumors obtained after subcutaneous coinjection was checked, suggesting that promotion of tumor development by miR-409 can effectively be obtained through exosomal transfer of the miR from stromal cells to cancer cells. Whether stromal exosomes-mediated stimulation can also favor prostate cancer occurrence remains to be determined but the authors also demonstrated that orthotopic implantation of miR-409-expressing stromal fibroblasts in mouse prostate induced prostatic luminal expansion, epithelial atrophy, increased inflammation, and both epithelial and stromal hyperplasia (126).

Another study used menstrual stem cells (MenSCs), that are MSCs isolated from menstrual blood (128). These cells behave as multipotent cells with finely tuned angiogenic properties necessary for the hormonal cycle of the endometrium. The MenSCs were shown to produce exosomes that can be internalized by PC3 cells. This incorporation targeted specific changes in cultured PC3 cells including decreased expression of VEGF (measured at both RNA and protein levels and in the culture media) and decreased production of reactive oxygen species (ROS). The authors further demonstrated that MenSC-derived exosomes suppress the secretion of proangiogenic factors (such as HIF1-alpha) by PC3 cells in a ROS-dependent manner, leading to decreased hemoglobin content and vascular density within the prostate tumors and thereby a reduction of tumor growth (128).

The last study explored EVs derived from pre-osteoblasts (considering as non-mineralizing since they are enable to deposit calcium) and mature osteoblasts (mineralizing and obtained from pre-osteoblasts upon dexamethasone stimulation) (129). The two EV populations disclosed differential protein expression depending on mineralization and could be internalized by PC3 cells. In the prostate cancer cells, RNA profiling analyses suggested that EVs internalization significantly induced transcriptional changes specific of the mineralization status. Although different in their nature, both transcriptional programs induced by (pre)osteoblast-derived EVs converged on pathways involved in cell survival and growth, a result consistent with the observed increase in PC3 cell growth under stimulation by the two EV populations (129).

### Effects of Cancer-Derived EVs on Prostate Cancer Cells

#### EV-Mediated Influence on Cell Behavior (Growth, Invasion, Migration, Plasticity)

Among cells that constitute the immediate microenvironment of a cancer cell are the neighboring cancer cells, on which exosomes can act as paracrine factors. If a specific intrinsic, genetic, or epigenetic, event occurs in a cancer cell and provide it a survival or growth advantage, it will be “vertically” transmitted to daughter cells. Exosomes are a means to transmit this advantage to “sister” cells, as a kind of “horizontal” transfer (130).

This phenomenon has been described for several cancers including the prostate cancer. Halin Bergstrom et al. (131) extracted EVs from the rat Dunning G (low-growing, androgen-sensitive, non-metastatic) and MML (rapidly growing, androgen-insensitive, metastatic) prostate cancer cell lines and injected them into the ventral prostate of naïve rats. Dunning G cells were then injected 72 h later at the same place. Tumors developed that were bigger when EVs were previously injected in rat prostates, in relation with a higher macrophage infiltration but also with an increased proliferation of rat normal epithelial cells (131). In other studies, normal epithelial prostate cell lines RWPE-1 and PNT-2 and prostate cancer cell lines LNCaP and DU-145 were exposed to EVs derived from the supernatants of primary cultures of PCa and BPH tissues as well as EVs extracted from plasma of PCa patients and healthy controls (132) or to exosomes extracted from LNCaP and DU-145 cells (133). Significant changes were observed in treated cells such as decreased apoptosis, increased migration and proliferation, increased secretion of interleukin-8 [a proinflammatory chemokine associated with the promotion and progression of several cancers (134)], alterations in gene expression suggestive of EMT (132, 133). Of note, observed effects differed according to both parental and target cells: LNCaP-derived exosomes had lower effect than DU-145-derived exosomes, while apoptosis was only reduced in LNCaP and proliferation was only increased in LNCaP and DU-145 cells and not RWPE-1 cells (133).

Integrins are membrane proteins necessary for cell-to-cell interactions and cell–cell junctions. Epithelial cells express integrins, whether they are malignant or not and prostate cancer cells do not escape this rule. Integrins can be found in the exosomes of prostate cancer cell lines. It has been demonstrated that integrins α_υ_β_6_ and α_υ_β_3_ are present in exosomes extracted from PC3 cells (and α_υ_β_3_ from CwR22 cells) (135, 136). They can be internalized by other cancer cells (DU145, C4-2B) that do not express these integrins, in which the proteins are then detected, without any change in their mRNA counterparts. A direct horizontal transfer of an immediately active biological molecule is, therefore, suggested that confers to recipient cells an increased ability to adhere and migrate (135, 136). Of interest, exosome-mediated transfer of α_υ_β_3_ could also be observed into non-malignant BPH-1 prostate epithelial cells (136). Whether the presence of these integrins could participate in prostate cancer progression and metastatic spread is even suggested by the demonstration of expression of α_υ_β_3_ in circulating exosomes extracted from the plasma of TRAMP mice (136).

CD9 is a member of the transmembrane 4 superfamily, also known as the tetraspanin family, and is considered as a key marker of exosomes. Soekmadji et al. recently demonstrated that exposure of LNCaP and DUCaP to dihydrotestosterone (DHT) increased the secretion of CD9-expressing exosomes (without any increase in the total number of secreted exosomes) (137). Proteomic analyses showed that DHT treatment, culture in androgen-free medium and treatment with the antiandrogen enzalutamide are effectively able to modify exosomes contents (137, 138). When CD9-enriched exosomes obtained after DHT treatment were inserted in the culture media of androgen-deprived LNCaP cells, an increased proliferation was observed. Whether this phenomenon could be observed *in vivo* as a means for cancer cells to escape or reduce the effects of androgen deprivation remains to be determined (137). It was recently demonstrated that AR can be loaded in EVs from LNCaP and 22Rv1 cells and thereby transferred to AR-null PC3 cells (139). In these recipient cells, AR proved to be functional through translocation to the nucleus and activation of target genes such as the PSA one (139). Of interest, EV-derived nuclear AR stimulated the proliferation of recipient cells in the absence of androgen, suggesting a new possible mechanism of resistance to androgen deprivation.

The interplays between AR and exosome pathways has been also highlighted in a recent experiment that used a variant of 22Rv1cells (140). Obtained by stable expression of the CRIPTO protein (an epidermal growth factor-related protein), this variant named Mes-PCa cells displays mesenchymal features that are accompanied by an increased released of EVs when comparing to parental cells. Androgen-dependent VCaP cells exposed to Mes-PCa-EVs showed decrease in both AR expression and transcriptional activity, activation of TGF-beta pathway, features of EMT, increased migrative, and invasive abilities as well as resistance to enzalutamide. Mes-PCa-EVs were found to express miRs known to target AR such as miR-21, miR-31 and miR-145 (140). EV-mediated cell plasticity, as observed for EMT, has also been described for NED (141). Although NED has frequently been reported as a result of androgen deprivation, the precise underlying mechanisms are still poorly understood (121, 122). Among soluble factors (cytokines, neuromediators, etc.) potentially involved, Il-6 proved to induce NED in PCa cells in association with lipid accumulation and induction of PPARgamma and adipocyte differentiation-related protein (ADRP) (141). In this study, C4-2B cells treated by Il-6 produced exosomes enriched in ADRP and able to be internalized by non-treated cells. Dendrite-like expression was, therefore, observed, both in PCa cells (DU-145 and C4-2B) and normal RWPE-1 cells. Of note, similar release of ADRP-containing exosomes were observed for cells treated by enzalutamide, confirming the potential induction of NED by androgen-deprivation therapy (ADT) (141).

It is unknown whether the exosome-mediated interactions between cancer cells could be used as a therapeutic tool (see below). Two independent studies nevertheless demonstrated that the oncosuppressor PTEN is loaded into exosomes and can thereby be transferred from PTEN-positive cells to PTEN-negative cells (142, 143). Moreover, they consistently disclosed decreased cell growth upon PTEN-containing exosome intake.

#### Horizontal Transfer of Chemoresistance From Cancer Cells to Others

Association between resistance to chemotherapy and EVs shedding by cancer cells was first experimentally suggested by Shedden et al. (144): a correlation was found between chemosensitivity to anticancer agents and expression of membrane shedding-related genes. Anticancer drug expulsion *via* shed vesicles was suggested since doxorubicin accumulated in vesicles to an amount higher than its cytoplasmic concentrations (144). More recently, Kosgodage et al. consistently demonstrated an increase in 5-fluorouracyl-mediated apoptosis of PC3 cells in the presence of two inhibitors of EV release (chloramidine and bisindolylmaleimide-I) (145).

More interestingly, EVs are now also thought to transfer resistance to chemotherapy. Several research teams established prostate cancer cell lines resistant to doxorubicine (146), camptothecin (147), docetaxel (148), or paclitaxel (149). The pioneer experiments showed that exosomes obtained from DU145 cells resistant to doxorubicine were able to confer chemoresistance to parental DU145 cells but also to LNCaP and 22Rv1 cells (146). Similar results were obtained with exosomes from doxorubicine-resistant 22Rv1 cells (146). An increased expression of p-glycoprotein (multidrug resistance MDR-1, or p-gp) was observed in the exosomes of resistant cells, confirming the hypothesis that exosomes could behave, under chemotherapy conditions, as an efflux means for anticancer agent (144). Similar results were observed with camptothecin with decreased apoptosis when exposition to exosomes from resistant cells (147). Similarly, even if exosome-mediated transfer of chemoresistance was not strictly observed, uptake of exosomes extracted from DU145 cells resistant to docetaxel by parental sensitive DU145 cells proved to induce an increase in anchorage-independent growth (soft agar colony formation) (148). It even seems that chemoresistance could be transferred by exosomes from malignant epithelial cells to stromal fibroblasts (103).

The molecular mechanisms that underlie exosome-mediated transfer of chemoresistance remain to be fully understood but exposition to chemotherapy expectedly proved to induce changes in the exosomal contents, as evaluated both for individual proteins or miR (146, 150, 151) and by high-throughput analyses (152, 153). For example, exosomes from taxane-resistant PC3 cells contain integrin β4 and vinculin in an extent similar to parental cells but to a higher amount as compared to exosomes from sensitive cells (150). Transcriptomic analyses of the culture media of DU145 cells treated by fludarabin showed a global increase in the miR production (153). In surviving cells, expression of miR-485-3p was found to be enhanced, leading to a downregulation of the transcriptional repressor NF-YB and the subsequent overexpression of genes involved in chemoresistance and/or survival, such as topoisomerase IIa, p-gp, and cyclin B2 (153).

Of interest, impaired sensitivity to chemotherapy can be reversed: when DU145 cells resistant to camptothecin were exposed to exosomes from the benign prostate cell line PrEC, anchorage-independent growth decreased (147). Similar results were observed for DU145 cells resistant to paclitaxel and exposed to exosomes from the benign prostate cell line RWPE (149). Sensitivity to paclitaxel was even partly recovered (149). It is worthy to note that the same effect was reported when resistant DU145 cells were exposed to exosomes from the human mesenchymal stem cells, a non-prostatic cell line (149), a result reminiscent of the controlling role of stroma on cancer cell growth. Proteomic analyses can be performed to explore the molecular pathways that sustain the phenotypic shift between induction and reversal of chemoresistance (147). Exosomes extracted from the sera of prostate cancer patients submitted or resistant to chemotherapy proved to decrease sensitivity to chemotherapy in naïve prostate cancer cell lines (146). Whether these fundamental researches could be translated in routine practice remains to be determined but consistent data suggest that exosome-mediated chemoresistance could be efficient *in vivo*.

#### Exosomes, Radiation Therapy, and Bystander Effect

Radiation therapy is one of the recommended treatment for localized PCa. It has been demonstrated that it acts by inducing premature senescence rather than apoptotic cell death (154). This premature senescence is associated with an increase in exosome secretion (155–157). Activation of exosome secretion in irradiated cells is regulated by TSAP6 protein (transmembrane protein tumor suppressor), which belongs to the p53 pathway (157, 158). The composition of exosomes is also modified by radiation. Irradiated prostate cancer cells have been shown *in vitro* to produce exosomes with elevated levels of B7-H3 (CD276) (155). Similarly, levels of exosome-derived Hsp72 were found elevated in the sera of 13 patients undergoing radiation therapy for PCa (159). Of note, tissue expression of B7-H3 has been linked to AR pathway and immune response (160) and evaluated as a prognostic or theranostic marker of PCa (160–164). Radiation therapy does not only influence exosome release, it also influences exosome internalization by recipient cells: it has been demonstrated that radiation of human bone marrow-derived MSCs induces an increased uptake of exosomes (165).

Secreted exosomes proved to influence the behavior of recipient cells as demonstrated in glioblastoma: irradiated cells secrete exosomes enriched in molecules involved in cell migration such as Connective Tissue Growth Factor and Insulin-like Growth Factor-Binding Protein 2 (166). After exosome incorporation into recipient cells, proteins are activated that are also involved in cell migration such as Src (166, 167), which has been shown to be enriched in prostate cancer cell exosomes (168). An interesting issue is the fact that effects of radiation are not limited to irradiated cells but is also communicated to adjacent cells, a phenomenon known as the radiation-induced bystander effect (RIBE) (157, 169, 170). Observed RIBEs are either beneficial (senescence of non-irradiated cancer cells) or deleterious (side-effects on non-malignant cells) and can be observed in cells adjacent to irradiated cells or in distant cells. The underlying mechanisms are incompletely understood but are likely to imply soluble clastogenic factors such as exosomes (157, 169, 170). First studies about the exosome involvement in RIBE initiation came very recently (171–173). As a demonstrating example, exosomes were isolated from the supernatant medium of BEP2D cells cultures (normal human bronchial epithelial cell line) and the expression profile of miRs were compared between exosomes collected from 2 Gy-irradiated cells and from non-irradiated cells (174). Several miRs were found to be differentially expressed, including miR-7-5p. Exosomes containing miR-7-5p proved to be internalized by non-irradiated BEP2D cells which, therefore, entered in autophagy, by reduced expression of EGFR, a known target of miR-7-5p (174). A distant bystander effect was also demonstrated *in vivo* (175). After focal brain irradiation of mice, exosomes were obtained from irradiated astrocytes and found to express high levels of miR-7. Injected into the tail vein of non-irradiated mice, they were able to induce autophagy of lung tissues by directly targeting Bcl-2 (175). Other miRs may have similar effects, such as miR-145, which proved to sensitize PCa cells to radiation (176). Whether these recent advances in RIBE mechanisms would be translated in clinical practice to enhance both tumor cell kill and normal tissue protection (for example through blockade of exosomal secretion) remains to be determined.

## Diagnostic and Prognostic Value of Prostate Cancer EVs

Several high-throughput analyses comparing body fluids from prostate cancer patients and controls demonstrated that the cargo of EVs is specific for the parental cells and the conditions in which they produce them (44, 46, 48, 53, 54, 59). Exploring whether EVs could be used as new providers of potential cancer biomarkers is the next logical step. Many factors influence EVs shedding and a first approach can, therefore, consist in evaluating the sole enumeration of EVs as a diagnostic tool, as first described in melanoma (177). As an easily available body fluid, urine was soon used as a means to detect EVs (83, 178) and demonstration has been made that prior prostate massage enhance the efficiency of urinary EVs detection (50, 179, 180). Urine from prostate cancer patients was, therefore, characterized by elevated exosomes secretion (50, 69, 178) and a decrease was observed after cancer treatment such as ADT (178). In plasma, feasibility was rapidly demonstrated through both *in vivo* mice experiments and direct blood sampling in human patients. Large oncosomes were detected in the plasma of TRAMP mice as caveolin-positive large vesicles and their number was found to be higher in mice with lymph node involvement or lung distant metastases (85). In Humans, studies consistently confirmed that plasma EVs are more numerous in prostate cancer patients than in healthy volunteers or patients with benign prostate hyperplasia (47, 51, 181–185). Diminution was observed after radical prostatectomy (183). A correlation was also observed with cancer aggressiveness, metastatic spread, and/or Gleason score (181–183). African-American patients were also demonstrated to have significantly higher amounts of EVs in their plasma and sera than European-American patients and, within their EVs, higher amounts of inhibitors of apoptosis proteins such as survivin, XIAP, and cIAP-2 (186).

Besides EVs enumeration, a specific EV component can also be specifically measured to help prostate cancer diagnosis. Again, urines and blood were tested and all molecules present in EVs can be evaluated (lipids, proteins, RNAs, miRs, long non-coding RNAs, etc.). As an example, a high-throughput mass spectrometry quantitative lipidomics analysis recently showed significantly different levels of nine lipid species including phosphatidylserine and lactosylceramide (58). Among exosomal proteins evaluated for their potential diagnostic value, PSMA, PSA, PTEN, survivin, p-gp, claudin3, FABP5, gammaglutamyl transferase, EGFR, ITGA3, ITGB1, TM256, and TGM4-ADSV were found elevated in the blood (plasma or serum) or the urine from prostate cancer patients (25, 35, 36, 48, 53, 54, 142, 151, 178, 181, 187–189) (Table 2). More generally, specific posttranscriptional modifications can also be explored: assessment of N-glycosylation pattern from urine EVs showed for example a decreased in overall biantennary core-fucosylation in prostate cancer patients (190).

**Table 2:** Clinical studies exploring exosomal proteins as potential prostate cancer biomarkers.

Protein (reference)	Origin of exosomes	Patients	Exosome isolation	Main results
*Claudin 3* (36)	Plasma	53 men with PCa 15 men with BPH 15 healthy controls	Differential centrifugation	Increased in PCa Gleason 8 vs BPH Increased in PCa Gleason 8 vs Gleason 6–7 No correlation with PSA

*FABP5* (54)	Urine	6 men with negative biopsies 12 men with PCa	Differential centrifugation	AUC = 0.757 for PCa diagnosis AUC = 0.856 for high-grade PCa (HGPCa) diagnosis^a^

*Gammaglutamyl transferase* (35)	Serum	31 men with PCa 8 men with BPH	Differential centrifugation	Increased in PCa

ITGA3 *ITGB1* (25)	Urine	5 men with lPCa 5 men with mPCa 5 men with BPH	Differential centrifugation	Increased levels in mPCa patients vs lPCa or BPH patients

*TM256* (48)	Urine	16 men with PCa 15 healthy controls	Differential centrifugation	AUC = 0.87 for PCa diagnosis AUC = 0.94 when combined to LAMTOR1

*Survivin* (187)	Plasma	47 men with PCa 20 men with BPH 16 healthy controls	Differential centrifugation	Increased in PCa vs BPH and healthy controls No correlation with Gleason score or recurrence after chemotherapy

*TGM4-ADSV* (53)	Urine	53 men with PCa 54 healthy controls	Differential centrifugation	AUC = 0.65

*CD63-GKPK5-PSA-PPAP-SPH* (53)	Urine	22 low-grade PCa^b^ 31 HGPCa^b^	Differential centrifugation	AUC = 0.70

Flotilin2 *Park7* (191)	Urine	26 men with PCa 16 healthy controls	Differential centrifugation	Specific detection using western blot and ELISA By western blot, flotilin2 distinguished between patients and healthy controls (AUC = 0.91) AUC felt to 0.65 using ELISA (and Park7 had a 0.71 AUC)

*PSMA* (181)	Plasma	82 men with PCa 28 men with BPH	Differential centrifugation and dextran gradient	Increased in PCa as compared to BPH AUC = 0.943; sensitivity = 83%; specificity = 92% Correlation with the Gleason score and the risk of biochemical recurrence No correlation with cT stage and tumor volume

*In all these studies, patients were classified as PCa or BPH according to the results of prostate biopsies. Healthy controls were volunteers without any prostate disease*.

*BPH, benign prostate hyperplasia; lPCa, localized PCa; mPCa, metastatic PCa*.

*^a^Low grade is defined as Gleason score ≤ 6 and high grade as Gleason score ≥ 7*.

*^b^Low grade is defined as Gleason score ≤ 7 (3 + 4) and high grade as Gleason score ≥ 7 (4 + 3)*.

PCA3 is a long non-coding RNA and has been proposed as urinary biomarker prostate cancer since 2003 (192). It has been detected in urine exosomes as soon as 2009 (83) and a specific assay based on the measure of PCA3 and ERG in urinary exosomes without prostate examination was developed to allow detection between Gleason score 6 vs Gleason score 7–10 prostate cancers (193). The real value of this specific exosomal test remains to be determined: although detection is more robust in the exosomes than in urine sediments (179, 180), it seems to be less efficient than the measure in the whole urine extracts (194). Another long non-coding RNA, lincRNA-p21, was identified in urine exosomes and appeared more abundant in prostate cancer patients than in patients with benign prostate hyperplasia (195). Other exosomal RNA species with suggestive diagnostic values are TMPRSS2:ERG fusion transcript, BIRC5, ERG, and TMPRSS2 (180, 196) (Table 3). In fact, a clear priority has been given to miRs because of fashion trend, because they represent the most abundant exosomal RNA species, and because exosome shedding and miR production are intrinsically related. Whether miRs circulate in blood or are present in body fluids (such as urines) freely (i.e., free of cells and cell compartments) or encapsulated in vesicles remains controversial, and this could depend on the nature of the body fluid itself (197–200). Because of this ambiguity, literature search using “EV” or “exosome” as keyword when looking for miRs as diagnostic tool for prostate cancer diagnosis dramatically reduces the number of published studies. Numerous papers reported detection and quantification of miRs in plasma or urines without any notion on whether the evaluated miRs were exosome-derived or not. We here focused on those papers where the exosomal origin was clearly specified while other reviews can be read to explore more largely the diagnostic value of miRs in prostate cancer (201–203).

**Table 3:** Clinical studies exploring exosomal RNA species as potential prostate cancer biomarkers.

Transcript (reference)	Origin of extracellular vesicles (EVs)	Patients	Exosome isolation	Main results
miR-141 *miR-375* (42)	Serum	47 men with recurrent PCa 72 men with non-recurrent PCa	ExoMiR extraction kit	miR-141 and miR-375 were associated with recurrent (metastatic) PCa following radical prostatectomy

*PCA3 and ERG (EXO106)* (204)	Urine	106 men with negative biopsies 89 men with positive biopsies	Urine Clinical Sample Concentrator Kit	Diagnostic value of the EXO106 score, the sum of normalized PCA3 and ERG RNA levels, in predicting biopsy results Prediction of PCa (Se: 75%, Spe: 54%) and prediction of high-grade PCa (HGPCa)

*PCA3 and ERG (ExoDx Prostate IntelliScore)* (193)	Urine	255 (training set) and 519 (validation set) patients subjected to biopsies	Urine Clinical Sample Concentrator Kit	Same assay than Exo106 Association of the score to standard of care (SOC) significantly better distinguished HGPCa (GS ≥ 7) from low-grade PCa (GS = 6) and benign disease than SOC alone Training set: AUC = 0.77 vs 0.66 Validation set: AUC = 0.73 vs 0.63

miR-1290 *miR-375* (205)	Plasma	23 men (screening cohort) and 100 men (validation) with CRPC	ExoQuick exosome precipitation solution	Correlation with overall survival

*lincRNA-p21* (195)	Urine	30 men with PCa 49 men with BPH	Urine Exosome RNA Isolation Kit	Significantly higher levels in PCa than in BPH (AUC = 0.663) No correlation with GS

*TMPRSS2:ERG* (196)	Urine	39 men with positive biopsies 47 men with negative biopsies	Filtration	Quantity of TMPRSS2:ERG transcript in patients with positive biopsies AUC was 0.744 for TMPRSS2:ERG AUC were 0.558 for AR, 0.674 for BirC5, 0.785 for ERG, 0.681 for PCA3

*PCA3* (194)	Urine	15 men with positive biopsies 14 men with negative biopsies	Centrifugation	Whole urine samples were more rich in PCA3 transcripts than exosomes Non-significant differences in exosomal PCA3 between patient with positive biopsies and those with negative biopsies

*miR-19b* (206)	Urine	20 healthy controls 14 PCa patients	Filtration	Mir-19b distinguished between PCa patients and healthy donors with a 100%-specificity and 93%-sensitivity (total urinary EVs) and a 95%-specificity and 79%-sensitivity (exosome-enriched subfraction)

*Isomirs of miR–21, miR-375 and miR-204* (207)	Urine	26 healthy controls 48 PCa patients	Centrifugation	Distinguished samples from control men and PCa patients than mature miRs The combination of the 3 isomirs with standards of care resulted in an AUC of 0.866, as compared to PSA (0.707) and the combination of the three corresponding mature microRNAs (miRs)

*miR-141* (208)	Serum	20 men with PCa 20 patients with BPH 20 healthy controls	ExoQuick Exosome Precipitation Solution	Higher levels in serum exosomes than in whole serum Higher levels in PCa patients vs BPH patients and healthy controls

*miR-141* (208)	Serum	51 men with PCa 40 healthy controls	ExoQuick Exosome Precipitation Solution	Higher levels in PCa patients vs healthy controls Correlation with PSA ≥ 10, GS ≥ 8, and T3/T4 stages Higher levels in mPCa vs localized PCa (AUC = 0.869)

*PSA, PCA3, ERG* (180)	Urine	12 men with negative biopsies 14 men with GS6 PCa 16 men with GS7 PCa	Ultrafiltration	Urinary exosomes were more rich in transcripts than cell pellets Detection of prostate-specific transcript including HoxB13, KLK2, PSA, PCA3, and ERG Increased PCA3 and ERG levels in PCa patients (no difference according to GS) No difference for PSA levels

miR-200c-3p miR-21-5p *Let-7a-5p* (209)	Plasma	24 men with PCa GS ≥ 8 26 men with PCa GS ≤ 6 22 men with BPH	Size exclusion chromatography	Plasma levels of EV miR-200c-3p and miR-21-5p were higher in PCa patients than in BPH patients (AUC = 0.38 and 0.67, respectively) Plasma levels of EV Let-7a-5p were lower in PCa GS ≥ 8 than in PCa GS ≤ 6 (AUC = 0.68)

miR-196a-5p *miR-501-3p* (210)	Urine	28 men with PCa 19 healthy controls	Centrifugation	Both miRs levels were decreased in PCa patients (AUC = 0.73 and 0.69, respectively)

*miR-2909* (211)	Urine	90 men with PCa 60 patients with bladder cancer 10 patients with BPH 50 healthy controls	Exiqon miRCURY™ exosome isolation kit	miR-2909 levels were only increased in urinary exosome from PCa patients miR-2909 levels correlated with GS

miR-21 miR-375 *Let-7c* (212)	Urine	60 men with PCa 10 healthy controls	Centrifugation	Levels of all three miRs are increased in PCa patients Combination of miR-21 and miR-375 can distinguish between the two groups (AUC = 0.872) Correlation of Let-7c with clinical T stage Correlation of the three miRs with PCa evolution risk

*miR-145* (213)	Urine	60 men with PCa 37 patients with BPH 24 healthy controls	ExoQuick Exosome Precipitation Kit	Urinary levels of exosomal miR-145 were increased in PCa patients vs BPH patients Higher levels if GS ≥ 8 as compared to patients with GS ≤ 7

*miR-1246* (214)	Serum	Various small cohorts	Total exosome isolation reagent (Life Technologies)	Serum levels of exosomal miR-1246 were increased in PCa patients vs BPH patients Higher levels in high stage PCa, in mPCA and CRPC

*BPH, benign prostate hyperplasia; CRPC, castration-resistant prostate cancer; GS, Gleason score; PCa, prostate cancer; mPCa, metastatic PCa*.

Several papers identified some exosomal miRs as potential diagnostic biomarkers of prostate cancer (Table 3). Among them, miR-141 is one of the most studied (in fact also for several cancers) because it belongs to the miR-200 family (along with miR-200a, b and c and miR-429). This family is involved in fundamental events of epithelial carcinogenesis such as cell transformation, EMT, or metastatic spread. In a cornerstone study about prostate cancer, miR-141 was identified (along with miR-375) among the exosomal miRs differentially expressed when comparing the expression of a panel of 742 miRs in plasma- or serum-derived circulating EVs in 78 prostate cancer patients and 28 healthy controls (42). Expression of miR-141 was found to be correlated with Gleason score and tumor progression, as well as the metastatic spread. Results in serum exosomes were independently confirmed (208, 215) while upregulation of miR-141 was also observed in urinary exosomes (52). Other exosomal miRs with potential diagnostic or prognostic values include miR-19b, miR-21, miR-34a, miR-375, miR-483-5p, miR-575, miR-1290, miR-1246, and let-7c (42, 52, 152, 205, 206, 212, 216). Serum exosomal let-7c has been correlated with Gleason score (212), miR-1290 and miR-375 to overall survival (205) while the decrease in plasma exosomal miR-34a was predictive of prostate cancer progression and poor response to docetaxel (152).

The situation in fact becomes more and more complicated. For example, recent studies deal with isomirs, the various miR isoforms generated through the miR processing and maturation process from a single miR locus (217). Koppers-Lalic et al. showed that the use of miR-21, miR-375, and miR-204 isomirs could provide a better diagnostic tool than that of the mature miRs (207).

Responses to ADT and to radiotherapy may also be predicted by the use of exosomal markers. A recent study suggests that some specific miRs in serum exosomes, particularly miR-654-3p and miR-379-5p, could be able to predict response to radiotherapy (218). As regards ADT, AR-V7 is an androgen receptor splice variant, which is constitutively active because of the truncation of the ligand-binding domain (219). The expression of AR-V7 constitutes a means by which prostate cancer cells escape to androgen-deprivation and Antonarakis et al. demonstrated a link between the expression of AR-V7 in prostate cancer circulating cells and resistance to the second generation antiandrogens enzalutamide and abiraterone (220). AR–V7 can be detected in plasma prostate cancer-derived exosomes (139, 221). Similarly to CTC-derived AR–V7, exosome-derived AR-V7 was found to be a reliable predictor of resistance to castration (221).

## Conclusion and Perspectives

As well as several cancers, a great hope emerged from studies demonstrating the role of exosomes, and more broadly EVs, in both prostate carcinogenesis, prostate cancer progression, and prostate cancer diagnosis. Numerous fundamental studies suggested that the various exosome contents could be used as a powerful tool to better understand how EV-mediated cell–cell interactions, as integral part of the reciprocal talk between malignant cells and their neighboring stromal cells, and constitute major events for cancer occurrence and progression. Numerous high-throughput analyses provided tens of proteins, RNAs, miRs as potential diagnostic and prognostic markers. Efforts are still warranted to translate these results into routine practice. Efforts have to be made to standardize EV classification and identification and to optimize the detection and isolation techniques. While the use of dedicated kits remains controversial (222), the development of specific and simple devices is, therefore, warranted (223, 224). Likewise, integrative approaches are needed that explored simultaneously the different exosomal contents. By exploring from the same samples both the miR and the long non-coding RNA repertories in prostate cancer cell lines, Ahadi et al. demonstrated that exosomal long non-coding RNAs are highly enriched in target motifs for the miRs that are also present in the same exosomes (37). This suggests that exosome sorting is an extraordinary organized process that allows selection of both specific biologically significant messages (miRs) and their specific conveyors (long non-coding RNAs).

Implication of exosomes in the prediction of the response to treatment (146, 152), in the prostate cancer prognosis or the patients’ overall survival (205), is highly promising of the announced shift toward personalized medicine. Whether exosomes could serve by themselves as therapeutic agents is also suggested (5, 225, 226). Their use as vectors of anticancer agents is advocated because they are cell-type specific (specific of both the parental and the target cells), lipophilic, and non-immunogenic by themselves, because they can cross membranes (including the blood–brain barrier), are not filtered by the glomerulus (long life in the circulation) and can carry very diverse molecules (proteins, lipids, RNAs, miRs, siRNAs, etc.) from diverse compartments (cell surface, cytosol) (227). EVs as transporters of anticancer agents have, therefore, been evaluated in prostate cancer although preclinical studies remain necessary before clinical applications (228). EVs can even function as cell-free vaccines to decrease or stabilize tumor growth (229). Since exosomes are able to significantly participate to cancer local progression and distal spread, it is also tempting to develop strategies able to block exosome-mediated procarcinogenetic effects: efforts are currently made to either block exosome release by parental cells, exosome circulation in the bloodstream and exosome interactions with target cells (125).

## Author Contributions

The author confirms being the sole contributor of this work and approved it for publication.

## Conflict of Interest Statement

The author declares that the research was conducted in the absence of any commercial or financial relationships that could be construed as a potential conflict of interest.
